# Highly active antiretroviral treatment for the prevention of HIV transmission

**DOI:** 10.1186/1758-2652-13-1

**Published:** 2010-01-12

**Authors:** Reuben Granich, Siobhan Crowley, Marco Vitoria, Ying-Ru Lo, Yves Souteyrand, Christopher Dye, Charlie Gilks, Teguest Guerma, Kevin M De Cock, Brian Williams

**Affiliations:** 1 Department of HIV/AIDS, World Health Organization, Geneva, Switzerland; 2 HTM, World Health Organization, Geneva, Switzerland; 3 UNAIDS India, Delhi, India; 4 Centers for Disease Control Kenya, Kenya; 5 South African Centre for Epidemiological Modelling and Analysis, Stellenbosch, South Africa

## Abstract

In 2007 an estimated 33 million people were living with HIV; 67% resided in sub-Saharan Africa, with 35% in eight countries alone. In 2007, there were about 1.4 million HIV-positive tuberculosis cases. Globally, approximately 4 million people had been given highly active antiretroviral therapy (HAART) by the end of 2008, but in 2007, an estimated 6.7 million were still in need of HAART and 2.7 million more became infected with HIV.

Although there has been unprecedented investment in confronting HIV/AIDS - the Joint United Nations Programme on HIV/AIDS estimates $13.8 billion was spent in 2008 - a key challenge is how to address the HIV/AIDS epidemic given limited and potentially shrinking resources. Economic disparities may further exacerbate human rights issues and widen the increasingly divergent approaches to HIV prevention, care and treatment.

HIV transmission only occurs from people with HIV, and viral load is the single greatest risk factor for all modes of transmission. HAART can lower viral load to nearly undetectable levels. Prevention of mother to child transmission offers proof of the concept of HAART interrupting transmission, and observational studies and previous modelling work support using HAART for prevention. Although knowing one's HIV status is key for prevention efforts, it is not known with certainty when to start HAART.

Building on previous modelling work, we used an HIV/AIDS epidemic of South African intensity to explore the impact of testing all adults annually and starting persons on HAART immediately after they are diagnosed as HIV positive. This theoretical strategy would reduce annual HIV incidence and mortality to less than one case per 1000 people within 10 years and it would reduce the prevalence of HIV to less than 1% within 50 years. To explore HAART as a prevention strategy, we recommend further discussions to explore human rights and ethical considerations, clarify research priorities and review feasibility and acceptability issues.

## Introduction

The trajectory of the beginning of the modern era of public health can be traced from Jenner's inoculation of James Phipps in England in 1796 to the last case of wild smallpox in Somalia in 1977. This is not to suggest that the focus of this commentary is the eradication of HIV, but rather that HIV is a virus and that we should visualize, as Thomas Jefferson did when he wrote to Jenner in 1806 that "future generations will know by history only that this loathsome disease has existed". Jefferson did not know that it would take 170 years before this would take place, but as the principal author of the Declaration of Independence, he was very familiar with hubris and taking the long view. Highly active antiretroviral treatment (HAART) may prove to be one of the combination prevention interventions that can help us to realize our collective goal of relegating HIV to the history books.

It is important to pause and consider the devastating extent of the HIV pandemic. In 2007, an estimated 33 million people were living with HIV [1]. About 68% reside in sub-Saharan Africa, with 35% in eight countries alone [1]. HIV is the strongest risk factor for tuberculosis (TB), and in 2008, there were about 1.4 million HIV-positive TB cases, representing 15% of global TB incidence [2]. About 26% of global TB deaths were estimated to be HIV associated, and 23% of HIV deaths were likely from tuberculosis [2]. In 2005, representatives of the G8 countries met in Scotland and committed to achieving universal access to HIV prevention, care and treatment by 2010 [1]. More recently, the G8 in Italy renewed its commitment. However, universal access remains a dream for millions of people and faces serious technical, economic and political challenges on a number of fronts [1].

Although there has been unprecedented investment in confronting HIV/AIDS - the Joint United Nations Programme on HIV/AIDS (UNAIDS) estimates $13.8 billion was spent in 2008 [3] - one of the key challenges facing us is not only how to sustain, but also to expand our response to the HIV/AIDS epidemic. This challenge is compounded by the fact that we are now facing the worst economic crisis since the 1930s. The magnitude and complexities of the economic problem are at times beyond comprehension. For example, the US lost more than 460,000 jobs in June 2009 alone and has already borrowed a trillion dollars. Many US states are nearly bankrupt and some have begun issuing paper credits in lieu of tax returns and salaries. The ripple effects of the US economic meltdown are being felt worldwide and is impacting investment in international and national public health.

We have made significant progress in meeting Universal Access objectives, and by the end 2008, there were 4 million people on HAART, almost 73% in Africa; 285,000 children were on HAART, a 45% increase since 2007 [4]. This is an extraordinary achievement that has required an unprecedented partnership of many stakeholders, combined with innovative approaches to resource mobilization. The expansion of access to HAART faced considerable skepticism by experts and others, who posited that treatment for HIV could not be delivered to impoverished countries in Africa and elsewhere. Service delivery for millions of people living with HIV is achieved one patient at a time and involves remarkable efforts by health care workers and people living with HIV and their families.

## Discussion

### The prevention gap

Despite these gains, there is an urgent need for both increased investment and more efficient use of available funding. Although 4 million people were on treatment by the end of 2008, by the end of 2007, 6.7 million needed treatment and in 2007, there were 2.7 million new infections. Around 23 million people were waiting, mostly unknowingly, to become treatment eligible, sicken or die. Although the additional 1 million people added on treatment in 2008 is impressive, with 2.7 million new infections in 2007, the treatment gap, which estimates the number of people with HIV eligible for HAART against those with access to HAART, only decreased from 95% to 69% by the end of 2007 [1]. We will need to dramatically reduce HIV incidence to keep pace with demand, with universal access appearing increasingly remote.

More troubling yet is the fact that these coverage percentage estimations are based on eligibility for HAART of CD4 counts of less than 200 cells/mm^3^, and will likely be revised *downward *to reflect starting treatment at higher CD4 thresholds. *The Economist *raised these concerns regarding the significant prevention and treatment gap: "As a result, taxpayers are accumulating an indefinite - and indefinitely growing - responsibility for keeping people alive. Somehow, somebody has to work out how to stop the disease spreading." [5] This economic perspective clearly emphasizes that as we expand treatment without decreasing HIV transmission, it will become progressively more difficult to cover the increasing costs.

### Human rights-based approach is essential

The HIV epidemic has raised issues of equity and human rights in ways that other public health threats may not have received similar attention. A human rights-based approach to building a strong public health response to HIV/AIDS is essential. It is widely recognized by public health experts and others that coercion and other mandatory approaches to addressing the HIV epidemic often have perverse negative outcomes. Although challenging, programmes that engage the community as a meaningful partner in the design and implementation have a greater chance of success, particularly when the potential for stigma and human rights violations exists [6]. There is increasing concern that the existing stark economic disparities will further exacerbate human rights issues and widen the increasingly divergent approaches to HIV prevention, care and treatment that are seen between rich and poor countries [7,8].

### Re-examining our current approach to controlling HIV

More than 27 years after the discovery of the HIV [9], control of the HIV epidemic remains elusive, and there have been calls to re-examine the current approach to controlling HIV. Given the clear HIV prevention gap, we must collectively find solutions to one of the most pressing public health problems facing us: how do we interrupt HIV transmission and drive the epidemic into the ground? Combination prevention includes a broad systemic analysis and tailored response that includes evidence-based interventions to address behavioural change, HAART, other biomedical strategies, and structural social justice and human rights interventions [10,11]. The rest of the article will focus on biomedical prevention interventions, but we need to keep in mind the importance of using our entire evidence-based arsenal in a combined prevention approach.

### Biomedical interventions

Research on biomedical interventions to interrupt sexual transmission have been disappointing, although there have been some signs of hope. Of 26 randomized controlled trials of different interventions, including four vaccine, 10 microbicide and three herpes suppression trials, 22 failed to show efficacy [12-14]. The four positive trials included three on male circumcision, and the sexually transmitted infection intervention trial in Mwanza, Tanzania, more than a decade ago, of limited generalizability [14].

At the Conference on Retroviruses and Opportunistic Infections (CROI 2009) in Montreal, Canada, in February 2009, Karim and colleagues presented results from HPTN 035 assessing the microbicide gel, Pro 2000 [15]. Though not reaching statistical significance, a 30% reduction in HIV incidence was observed in gel users. Pre-exposure prophylaxis (PrEP) can be provided through topical, as well as oral antiretroviral agents [15]. Encouraging animal data suggest that a HAART-containing gel may provide pre-exposure protection [16]. The efficacy of oral PrEP is being assessed in 10 ongoing or planned randomized controlled trials involving some 20,000 participants internationally; the first results are expected in late 2009 or 2010. Although PrEP is promising, it may take unusual persuasiveness to convince a decision maker to give drugs to HIV-uninfected persons when many with declared HIV disease are dying from lack of access.

Recent results from the vaccine trial in Thailand [17] remind us that while implementing evidence-based, near-term prevention solutions, work must continue to develop a vaccine that could be used in the future to mitigate the HIV epidemic. The overall situation has prompted many people to consider the potential prevention role of HAART.

### Scientific evidence for HAART as prevention

Before considering the potential impact of HAART for prevention, it is important to consider the scientific evidence for the basic assumptions behind HAART for prevention. These assumptions include the obvious but often overlooked fact that HIV transmission occurs only from people with HIV [9].

There are studies supporting the assumption that viral load is the single greatest risk factor for all modes of transmission. Quinn's landmark study in Rakai, Uganda, showed that patients with less than 400 copies of HIV RNA per millilitre. have the lowest rate of HIV transmission, and demonstrated a stepwise increase in transmission rates for higher RNA levels [18]. Lowering viral load is essential to interrupting transmission, and HAART can lower viral load to nearly undetectable levels. A 2009 meta-analysis that included 11 cohorts and 5021 heterosexual couples concluded that there was zero risk of sexual transmission while on HAART for those with HIV-1 RNA below 400 copies with an upper confidence limit of 1.27 per 100 years [19].

Prevention of mother to child transmission (PMTCT) offers proof of the concept of HAART interrupting HIV transmission. Although some would argue that lessons from PMTCT may not be applicable to preventing sexual transmission, perinatal AIDS cases have been virtually eliminated in the United States [20]. This is likely due to the implementation of Public Health Service guidelines for the universal counselling, voluntary HIV testing and HAART for pregnant women and newborn infants [20]. Recent trial data from sub-Saharan Africa support HAART to block PMTCT, with one study showing a decrease of transmission to 1% [21].

Observational studies illustrate the potential for HAART for prevention of HIV transmission [22]; Bunnell and colleagues in Uganda showed that when HAART is added to couples counselling, transmission during sex can be reduced by 98% [23]. Numerous studies suggest a potential for the community-level impact of HAART on HIV transmission. In British Columbia, public health scientists showed a decrease in community plasma HIV RNA concentrations and HIV incidence among injecting drug users associated with HAART use [24]. Work from Taiwan found a 53% reduction in new HIV cases associated with the provision of free access to HAART [25]. Additional data from areas with high antiretroviral treatment (ART) coverage do exist, and additional community-based studies examining the impact of HAART on HIV transmission are in preparation.

### When to start?

Although knowing one's HIV status is key for prevention, it is not known with certainty how early to start HAART. People living with HIV in sub-Saharan Africa start HAART at a median CD4 count of around 100 cell/mm^3^, which is much later than in the north [26]. This lack of access to HAART until very late in the course of the disease is decreasing in areas as people learn their HIV status earlier, guidelines change and services expand. However, mortality is still markedly higher in sub-Saharan Africa compared with other contexts [26,27].

At CROI 2009, Lawn and colleagues from South Africa showed a steeply increasing risk of death in patients on HAART associated with the time spent below 200 CD4+ cells/mm^3 ^[28,29]. Mortality reached almost 40/100 person years when the CD4+ count was less than 50 [28,29]. Although mortality rates at the higher CD4 levels may be relatively low, when applied to large numbers of people living with HIV, this lower risk converts into a significant impact on mortality. Observational data from North American cohorts showed that among 8362 patients with a CD4+ count of 351 to 500 cells/mm^3^, deferral of therapy until the CD4+ count had fallen to 350 cells/mm^3 ^or less was associated with an increase of 69% in the risk of death, as compared with patients who initiated therapy when their CD4+ count was within the designated range (relative risk in the deferred-therapy group, 1.69; 95% confidence interval [CI], 1.26 to 2.26; P < 0.001) [30].

Similarly, among 9155 patients with a CD4+ count of more than 500 cells/mm^3^, deferral of therapy until the CD4+ count fell below 500 cells was associated with a significantly increased risk of death of 94% (relative risk, 1.94; 95% CI, 1.37 to 2.79; P < 0.001) [30,31]. Although not a randomized clinical trial and relying on relatively few events, it nevertheless presents further evidence for the potential benefits of starting earlier and is in line with other observational data [26]. Sax and Baden, in their accompanying editorial, suggest that we may be moving to an era when most people will choose to start HAART when they are ready [31]. The When to Start Consortium analyzed data from 18 cohorts in Europe and North America and included more than 40,000 patients [26]. Starting treatment at higher CD4 counts reduced the probability of AIDS or death, with those starting before reaching a level of 450 cells/mm^3 ^having the most benefit [26]. There are numerous other cohort studies that also suggest that starting earlier is better [32-34].

Trial data, although limited, also point in the direction of an earlier start. The National Institutes of Health-supported randomized clinical trial, CIPRA HT 001, in Haiti was stopped by the Data Safety Monitoring Board. Patients who started treatment earlier, with CD4 counts of between 200 and 350 cells/mm^3^, had significantly fewer deaths and fewer cases of TB compared with those who had deferred treatment to less than 200 cells/mm^3 ^or when an AIDS-defining illness occurred.

ACTG A 1564, conducted predominantly in the United States, compared early with deferred HAART in patients treated for various opportunistic events (but not TB) [35]. Survival curves show a 47% reduced progression or death in patients receiving immediate as opposed to deferred HAART [35]. There is increasing evidence that suggests the damaging effects of HIV, even at higher CD4 count levels [36,37]. The SMART trial showed not only that starting earlier provided superior outcomes; it also suggested that HIV may be associated with serious non-AIDS-defining events, including cardiovascular, renal and liver disease and non-AIDS malignancies [36,37]. For some, this evidence of HIV infection as a chronic inflammatory disease process provides additional rationale for an earlier start of HAART.

TB is the major killer for most people living with HIV in sub-Saharan Africa. Studies from Cape Town demonstrate increasing TB incidence, and the group has coined the phrase, "TB death zone", to describe living below 500 cells/mm^3 ^[28,29]. There is increasing recognition among people working on HIV-related TB that HAART has a significant role to play in preventing TB morbidity, transmission and mortality. Preliminary results of the South African SAPIT trial, presented at CROI 2009, compared outcome of HAART integrated with TB treatment versus deferred treatment until TB therapy was completed in patients with CD4+ counts below 500 cells/mm^3 ^[15]. There was a 56% reduction in mortality in the integrated group, and this applied across the whole CD4+ spectrum in a stratified analysis [15].

The emerging evidence suggests that HAART should be initiated as soon as possible in acute illness. The evidence increasingly suggests that if the future is to be different, we have to intervene with treatment far earlier, before people living with HIV fall into or spend too long in the CD4+ "death zone" for tuberculosis.

Under universal access, most people living with HIV will eventually become eligible and require HAART. How early to start HAART is a matter of perspective, and the crux of the issue is time from HIV infection. A recent study gathered CD4 and RNA data from 30 international studies and 16 cohorts of untreated adults with HIV [38]. The analysis of median CD4 counts over time found relatively low starting levels and a relatively rapid progression to commonly discussed CD4 thresholds, such as 500, 350 and 200 [38].

The time to reach commonly used CD4 eligibility thresholds for HAART was variable and in some settings, only a few years after HIV infection [38]. Data suggests that on average it takes about two to four years to reach a CD4 count of 500 cells/mm^3^, depending on one's starting point; from this perspective this represents a significant amount of time. However, if we consider that people will live for tens of years on HAART, then the two to four years it would take to reach a CD4 level of 500 cells/mm^3 ^makes up only a small fraction of the remaining years of life. In other words, optimally most people will have access to HAART, and when discussing whether to start at 200, 350 or 500, we are really discussing the difference of a few years earlier in the course of a much longer life span on HAART.

Guidelines are written for the context within which they are meant to be applied, and European, North America and World Health Organization (WHO) recommendations are divergent on a number of important issues. In addition to clear differences in formulary and laboratory support, in countries with more resources, people are started earlier, and factors, such as viral replication status, CD4 decline and being in a discordant couple, are now appearing as potential start criteria, even at higher CD4 counts.

Consideration of the durability of currently used regimens in resource-constrained settings will be central in evaluating using HAART for prevention. Our ongoing scientific and moral challenge will be to continue to narrow the treatment gulf between north and south and to ensure that we do not accept the establishment of two standards of care: one for the richer countries and the other for the poorer, for lack of better terms.

Essentially all people living with HIV will eventually require HAART for clinically progressive, ultimately fatal immunodeficiency. Regardless of when we decide that people should start HAART, we will not reach universal access to prevention, care and treatment unless millions of people with HIV learn their HIV status. Despite considerable efforts to expand access to HIV testing, an estimated 80% of people living with HIV in sub-Saharan Africa do not know their status and 90% do not know their partners' status [1].

Kenya is a leader in improving access for HIV counselling and testing. However, data from the 2007 Kenya AIDS Indicator Survey shows that of people eligible for HAART (with a CD4 count of less than 250), 57% have no idea that they have HIV [39]. Knowing one's HIV status was a key determinant in accessing treatment in Kenya: 92% of those who knew their status and were eligible were on HAART [39]. Susan Allen, working in the late 1980s and '90s in Rwanda and Zambia, and many others have built the scientific evidence base to show that HIV counselling and testing itself [23,40,41], particularly when it involves couples counselling, can be a remarkably effective prevention intervention [23,41,42].

Community-based efforts, including home-based couples counselling and testing, have considerable promise. Through a seven-day campaign designed to prevent HIV, diarrheal disease and malaria in a district in western Kenya, a private sector company working with local non-governmental organizations, Centers for Disease Control Kenya, and the Ministry of Health was able to test 41,040 or 80% of the men and women between 15 and 49. The total population reached was 47,311, out of which 97% were voluntarily tested and counselled for HIV. Of those reached, 18,300 or 38% were men [43].

Of course, knowing one's HIV status is not enough: the cornerstones of HIV counselling and testing scale up must include improved protection from stigma and discrimination, as well as improved access to integrated prevention, treatment and care services. A human rights approach, based on the "3 Cs" of HIV testing (confidentiality, counselling and informed consent) is a prerequisite for success [44].

### Modelling results

This quote from George Box, one of the most influential statisticians of the last century, puts it nicely: "Essentially, all models are wrong, but some are useful." Models help us to better understand what we think we know and perhaps most importantly, what we need to find out. Our model [45] builds on and extends earlier analyses suggesting that rapid scale up of conventional HAART approaches could significantly reduce mortality [46] and have a substantial impact on HIV incidence [47,48].

R_0 _or reproductive ratio is the average number of secondary cases of infection to which one primary case gives rise throughout its infectious period. We focused on a generalized HIV epidemic setting largely driven by heterosexual sex, and used data from South Africa, Uganda, Malawi and elsewhere [45]. The South Africa HIV surveillance data shows an initial doubling time of 1.25 years, which means that on average each person with HIV infects another person once every 1.25 years. Life expectancy for people with HIV was 10 years, which allows us to estimate an R_0 _of around 7. Therefore, we assumed that cutting transmission by a factor of more than 8 would reduce R_0 _< 1 and eliminate HIV infection.

We used a stochastic model for estimating R_0 _and we examined phases and relative infectivity with time. The parameter values for infectivity are not known precisely, but we used the calculated R_0 _and the literature for our assumptions. The relative importance of the acute phase and concurrency has been the subject of some debate among modellers, epidemiologist and others. We conducted sensitivity analyses by varying the degree of infectiousness and the duration of the acute, chronic and final phases [45].

We used a stochastic model to examine the frequency of HIV testing and CD4 level needed to get R_0 _< 1. We would need to test people on average about once per year and need to start them on immediate HAART when their CD4 count was around 1000. In the South African setting, the average CD4 count after conversion is 884. Therefore to reach R_0 _< 1 for most people, we need to start HAART immediately, irrespective of CD4 count. We also see that annual testing and a CD4 threshold of 200 gives you a R_0 _of 4 - a significant reduction in transmission but not elimination, which we defined as 1000 cases per million per year [45].

For the model, we used the 2007 estimate that 5% of people living with HIV were already on HAART and that programme coverage increases logistically. In other words, programme coverage reaches 50% in 2012 and 90% in 2016 [45]. The model's programme start date can be altered accordingly, with more rapid implementation translating into shortened time to maximal impact. We added in a 40% prevention effect whereby transmission would be additionally decreased by 40% over the time period. For some, this is optimistic for a combined prevention approach, and for others, not high enough, but we felt that if current prevention efforts beyond HAART could decrease transmission by 40%, that would be reasonable.

Although this combined approach dramatically reduces transmission, it does mean that a large cohort of people will be on HAART for a long time; of course, we thought that this was not a bad thing since they would at least be alive, and it is similar to accelerated universal access, but nevertheless represents a significant challenge. Modelling showed that the universal voluntary HIV testing and immediate HAART strategy with combined prevention interventions resulted in a 95% reduction in incidence or new HIV cases in 10 years. The theoretical strategy reduced incidence from 15-20,000 per million population to 1000 per million and the prevalence to less than 1% by 2050 [45].

The model suggests that mortality would decline rapidly and the epidemic would become concentrated with particular populations remaining at risk. Health services could then switch focus from making HAART available to those in greatest need to providing support and services for those who are on HAART. While other prevention interventions, alone or in combination, could significantly reduce HIV incidence, the model suggests that only universal HIV testing and immediate initiation of HAART could reduce transmission to the point where elimination might be feasible by 2020 for a generalized epidemic, such as that in South Africa. The less than 350 strategy has a major impact, but we are left with a persistent epidemic. In summary, the universal voluntary HIV testing and immediate treatment strategy drives down incidence, prevalence and mortality towards an elimination phase [45].

The different strategies do come with different impacts: the less than 350 on HAART strategy could save nearly 2.41 million lives, while the universal voluntary HIV testing combined prevention approach nearly triples that number to 7.35 million [45]. These dramatic results beg the question: how much will it cost? We did a rough costing for the strategies based on the estimated costs of delivering ART [45]. The funding needed to implement the theoretical strategy for an epidemic of South African-type severity peaks in 2015 at $3.4 billion per year (range: $2.2 billion-$5.3 billion).

Although the initial yearly cost of the theoretical strategy is higher than the present strategy, it is within UNAIDS projections of the $8.84 billion needed every year for universal access to prevention, care, and treatment in a South African-type situation in 2015 [45]. The "front-loaded" universal voluntary strategy is initially more expensive, but becomes cost saving around 2030 as the HIV epidemic moves toward elimination [45]. A programme of this size would require considerable initial effort. However, over time, the decreasing HIV incidence would free scarce health care resources that are currently overwhelmed by the immediate demands of the HIV epidemic.

### Next steps

The theoretical strategy of universal voluntary HIV and immediate HAART raises a new set of challenges and, as with all prevention interventions for HIV, this approach should not be viewed independently of other methods of prevention [49]. The strategy assumes relatively rapid programme expansion, high adherence, and significant community acceptance and participation. Expert evaluation and further research and discussion is required to assess this novel approach, its appropriateness and its feasibility, and to define the requirements for public health decision making on how to best use of HAART for prevention and control of HIV/AIDS.

We are engaged in further modelling on other important aspects, such as the impact of HAART on TB, the relative importance of drug resistance and other assumptions, the impact of PrEP, effects on PMTCT, and an in-depth economic analysis of the various strategies. Preliminary results are very interesting and we look forward to sharing them soon.

We hope that this work has helped key stakeholders to consider the potential role of HAART as part of combined HIV prevention efforts. In early November 2009, WHO held two meetings to discuss HAART for prevention [50]. As part of WHO's convening role, stakeholders were invited to explore human rights and ethical considerations, clarify research priorities and review feasibility and acceptability issues (the presentations, selected articles, list of participants and outcomes of the meeting are available at http://www.who.int/hiv/topics/artforprevention/.

## Conclusions

The model has raised many questions for all of us. We are reminded of this quote by Hermann Biggs, a pioneer in United States public health: "Public health is purchasable. Within a few natural and important limitations any community can determine its own health."

Unlike the weather, HIV is an infectious disease and can be controlled and possibly even eliminated: it will be up to us to work together to make that happen using the many tools that we have developed over the past 27 years. Although HAART, condoms, couples counselling and a future vaccine are critical tools, their true public health impact can only be achieved when we use our common purpose and ability to reason to address one of the toughest public health problems facing our global community.

## Competing interests

The authors declare that they have no competing interests.

## Authors' contributions

RG drafted manuscript based on the 2009 IAS Cape Town Plenary in collaboration with SC, MV, YL, YS, CD, CG, TG, KMDC, and BW. All authors contributed to writing of the report and have seen and approved the final version of the report.

## Disclaimer

The opinions and statements in this article are those of the authors and do not represent the official policy, endorsement or views of the World Health Organization.
